# New type of highly active chromium(III) catalysts containing both organic cations and anions designed for polymerization of beta-olefin derivatives

**DOI:** 10.1038/s41598-018-20665-x

**Published:** 2018-02-02

**Authors:** Joanna Drzeżdżon, Artur Sikorski, Lech Chmurzyński, Dagmara Jacewicz

**Affiliations:** Faculty of Chemistry, University of Gdańsk, Wita Stwosza 63, 80–308 Gdańsk, Poland

## Abstract

The new type of catalysts designed for the olefin derivatives polymerization has been synthetized. The novel catalysts are chromium(III) salt type complexes composed of both organic cation and anion, i.e. [Cr(dipic)_2_][Cr(bipy)(dipic)H_2_O]∙2H_2_O and [Cr(dipic)_2_]Hdmbipy∙2.5 H_2_O. The compositions of these complexes have been confirmed by a number of instrumental methods including NMR, IR, UV-Vis, MS and elemental analysis ones. Moreover, the crystal structures of these novel catalysts were determined and reported. Furthermore, the [Cr(dipic)_2_][Cr(bipy)(dipic)H_2_O]∙2H_2_O and [Cr(dipic)_2_]Hdmbipy∙2.5H_2_O complexes have been studied towards their catalytic activity, after the activation by MMAO (modified methylaluminoxane), in the case of 2-chloro-2-propen-1-ol polymerization at 21 °C and atmospheric pressure. It has been found that novel catalysts, [Cr(dipic)_2_][Cr(bipy)(dipic)H_2_O]∙2H_2_O and [Cr(dipic)_2_]Hdmbipy∙2.5 H_2_O, exhibit a very high catalytic activity in the process of the polymerization of the beta-olefin derivatives. The products of a such catalyzed polymerization are the poly(allyl alcohol) derivatives.

## Introduction

Polyolefins and their derivatives are commonly used in the world production of polymer materials such as polyethylene, polypropylene and poly(vinyl alcohol)^1,2^. The mass production growth of polyolefins is dependent on continuous technological advances in the field of catalysts in low-pressure (1–5 ∙ 10^5^ Pa) polyolefin production^3,4^. Organometallic complexes are used as α-olefin polymerization catalysts^4,5^.

Since Hogan and Banks^6^ discovered the catalytic activity of a silica support treated with CrO_3_ (the Phillips catalyst), the chromium(III, VI) complexes have become the subject of studies concerning the large-scale industrial production of polymer materials^3^. The chromium(III) complexes play a significant catalytic role in the commercial olefin polymerization^7^. These catalysts are activated by methylaluminoxane or its modified form (MAO or MMAO)^8,9^. The olefin polymerization catalysts include the metallocene complexes of chromium(III) which exhibit a high catalyst activity such as the amino-substituted cyclopentadienyl chromium(III) complex (8300 g∙mmol^−1^∙h^−1^∙bar^−1^ in the propylene polymerization). However, the greater part of chromium(III) metallocene complexes are unsatisfactory for an industrial application because they are unstable at high temperatures during polymerization processes. Moreover, the metallocene complexes after the reaction with MAO/MMAO decompose slowly. Thus, the non-metallocene complexes of chromium(III) with particular focus on non-cyclopentadienyl-based complexes are a new generation of catalysts for the olefin and its derivatives polymerization. The non-cyclopentadienyl complexes of chromium(III) contain the neutral and monoanionic ligands. The Cr(III) complexes containing neutral ligands exhibit a low or moderate catalytic activity, e.g. Cr[N(SiMe_3_)_2_]_2_I_2_ has 43 g∙mmol^−1^∙h^−1^∙bar^−1^ ^10^, chromium(III) complex with 2-(1-isopropyl-2-benzimidazolyl)-6-(1-(arylimino)ethyl)pyridines exhibit the 114 g∙mmol^−1^ ∙h^−1^∙bar^−1^ activity^11^. The another catalyst group constitute chromium(III) complexes with monoanionic ligands, for example bis(salicylaldiminato) chromium(III) complex having 96 g∙mmol^−1^∙h^−1^∙bar^−1^ catalytic activity^12^. Among the complexes cointaing tridentate monoanionic ligands bis(phosphino)amide complex of chromium(III) exhibits the 500 g∙mmol^−1^∙h^−1^∙bar^−1^ activity^13,14^. Furthermore, among the non-metallocene complexes the highest catalytic activity (6970 g∙mmol^−1^∙h^−1^∙bar^−1^) is observed for chromium(III) complex with triptycenyl and 2-pyridylmethyl^3^. This chromium(III) complex contains the tridentate [O,N,N] monoanionic ligand. However, it should be emphasized that in this case the polymerization product at 50 °C is the low-molecular-weight polyethylene (PE)^15,16^.

In this paper, for the first time, we report a new type of highly active catalysts for the olefin derivatives polymerization. The novel catalysts are chromium(III) organic salt type complexes composed both of organic cations and anions. They are based on dianionic tridentate ligand and N-heterocyclic compounds. Two complexes [Cr(dipic)_2_][Cr(bipy)(dipic)H_2_O]∙2H_2_O and [Cr(dipic)_2_]Hdmbipy∙2.5H_2_O (dipic - dipicolinate, bipy − 2,2′-bipyridine, dmbipy - 4,4′- dimethoxy - 2,2′- bipyridine) have been designed and synthesized. In the next step they were investigated as catalysts, after activation by MMAO (modified methylaluminoxane), in the case of the 2-chloro-2-propen-1-ol polymerization at 21 °C and at atmospheric pressure. We report herein the first example of the preparation of poly(2-chloroallyl alcohol) using the complex compounds as catalysts. These results give the prospect of the industrial use of the reported complexes as catalysts in the beta-olefin derivatives polymerization. It is of big importance since the polymers of the beta-olefin derivatives are used to the production of coatings or elastomers. The analysis of literature data shows that the novel complexes have about 5 times higher catalytic activity than the majority of non-metallocene chromium(III) catalysts used mainly to the propylene and ethylene polymerization. Moreover, [Cr(dipic)_2_][Cr(bipy)(dipic)H_2_O]∙2H_2_O and [Cr(dipic)_2_]Hdmbipy∙2.5H_2_O catalyze the polymerization at the room temperature. Furthermore, the synthesis of these compounds are simple, cheap and efficient. The products of the polymerization are the poly(allyl alcohol) derivatives. To sum up, this report shows the possibility of creating a new class of chromium(III) complexes, similar in their structure to the organic salts, as catalysts for the poly(allyl alcohol) derivatives polymerization.

## Results

### The structures of new complexes

**Figure 1:**
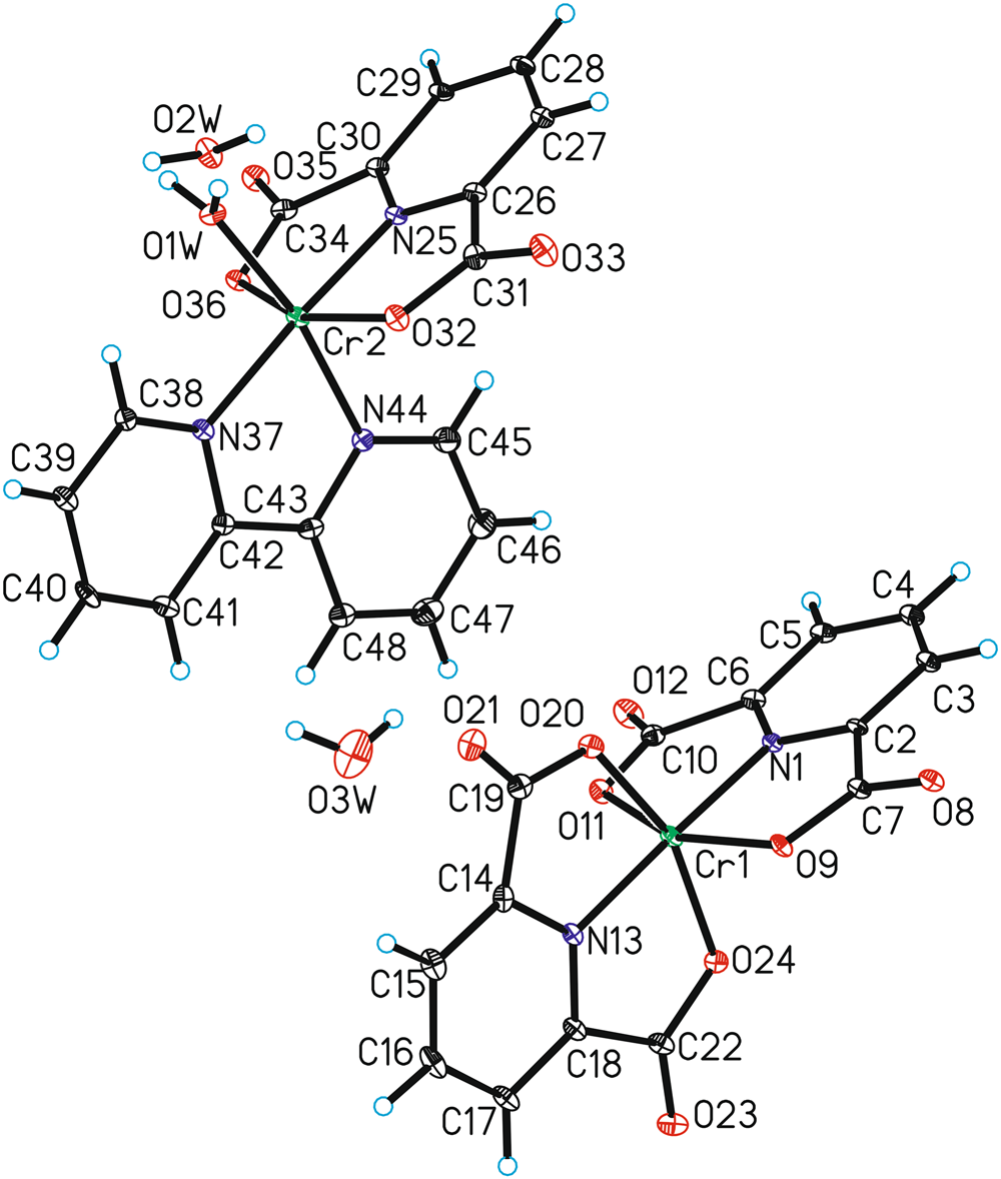
The molecular structure of [Cr(dipic)_2_][Cr(bipy)(dipic)H_2_O]∙2H_2_O. Displacement ellipsoids are drawn at the 50% probability level (disordered water molecules have been omitted).

**Figure 2:**
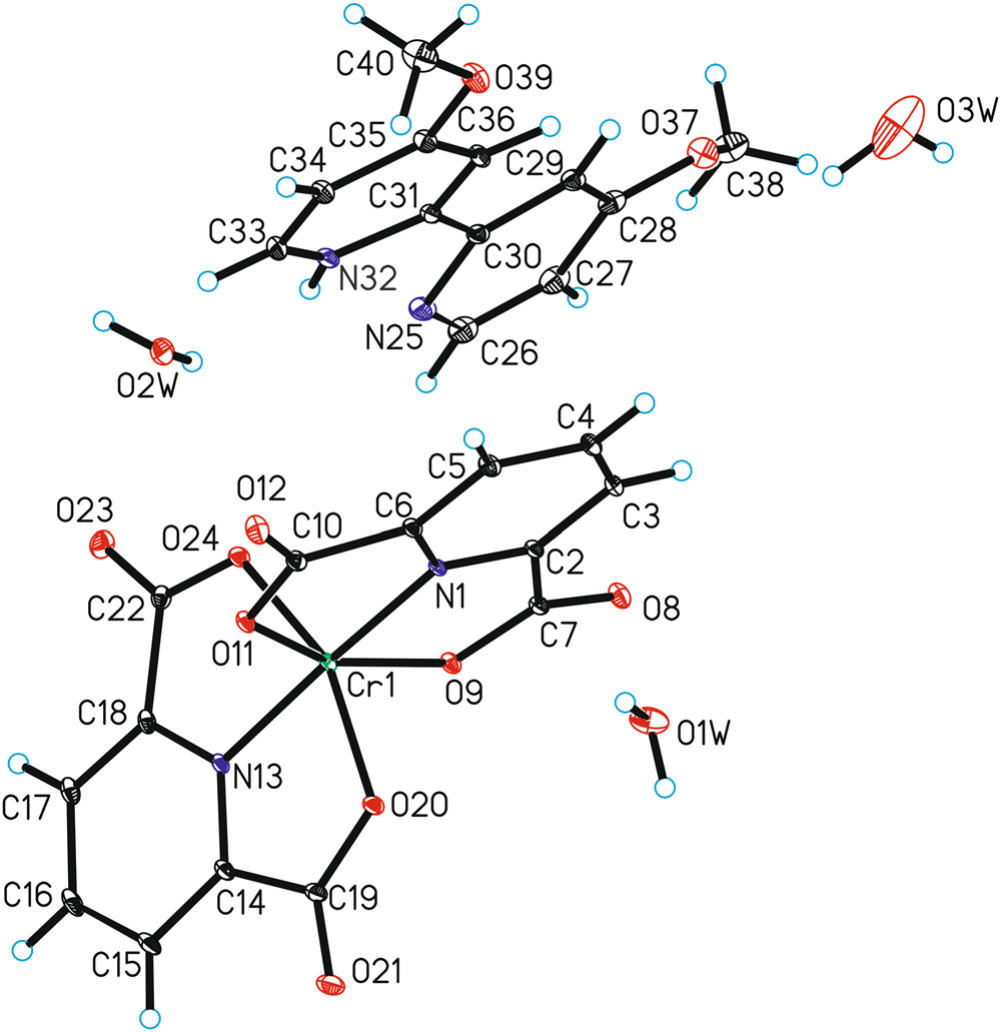
The molecular structure of Cr(dipic)_2_]Hdmbipy∙2.5H_2_O. Displacement ellipsoids are drawn at the 50% probability level (disordered water molecules have been omitted).

The crystal structures of the novel chromium(III) complexes - [Cr(dipic)_2_][Cr(bipy)(dipic)H_2_O]∙2H_2_O (Fig. 1) and [Cr(dipic)_2_]Hdmbipy∙2.5H_2_O (Fig. 2) have been determined by the X-ray diffraction method. The new polymorph of [Cr(dipic)_2_][Cr(bipy)(dipic)H_2_O]∙2H_2_O has been reported. In the studied crystal structure of [Cr(dipic)_2_][Cr(bipy)(dipic)H_2_O]∙2H_2_O the crystal packing of the anionic part is different compared to the crystal of another polymorphic variant of this complex^17^. In the crystal structure described in this report the tetramers are bonded by hydrogen bonds between H_2_O_coordinating_ and H_2_O_crystallization_. One oxygen atom from dipic included in ([Cr(bipy)(dipic)H_2_O]^+^) interacts with the crystallization water and the oxygen atom of the dipic from the [Cr(dipic)_2_]^−^ anion. In the crystals of [Cr(dipic)_2_][Cr(bipy)(dipic)H_2_O]∙2H_2_O, neighbouring ions are linked by π_(bipy)_ − π_(picoline)_ interactions and C–H···O hydrogen bonds to form tubes along the *b*-axis (see Supplementary Material). The adjacent tubes are linked *via* O–H···O and C–H···O hydrogen bonds between ions and water molecules forming a 3D-framework structure.

In the crystal packing of [Cr(dipic)_2_]Hdmbipy]∙2.5 H_2_O ions are linked via π_(dimethoxybipy)_ − π_(picoline)_ interactions and C–H···O hydrogen bonds to form blocks along the *c*-axis (see Supplementary Material). The neighbouring blocks are linked by N–H···O, O–H···O and C–H···O hydrogen bonds through water molecules forming a three-dimensional framework structure.

In addition, the synthesized novel complexes were subjected to full spectroscopic analysis, where the results are following:

1. [Cr(dipic)_2_][Cr(bipy)(dipic)H_2_O]∙2H_2_O
  *UV-Vis*: The regions of the maximum absorption occur at 421 nm and 551 nm. (in DMSO)
  ^1^*H NMR* (DMSO-d_6_, 500 MHz): δ 8.71 (ps, 2 H, 6,6′-arom. H from bipy), δ 8.42 (ps, 2 H, 3,3′-arom. H from bipy), δ 7.49 (ps, 2 H, 5,5′-arom. H from bipy), δ 8.24 (ps, 6 H, 3, 5, 3′, 5′-arom. H from dipic), δ 7.99 (ps, 3 H, 4, 4′-arom. H from dipic).
  ^13^*C NMR* (DMSO-d_6_, 125 MHz): δ 165.65 (3 C, 2,2′-arom. C from dipic), δ 155.21 (3 C, 4,4′-arom. C from dipic), δ 151.30 (3 C, 6,6′-arom. C from dipic), δ 125.00 (3 C, 3,3′-arom. C from dipic), δ 116.74 (3 C, 5,5′-arom. C from dipic), 165.93 (6 C, C=O), δ 169.05 (2 C, 2,2′-arom. C from bipy), δ 154.61 (2 C, 4,4′-arom. C from bipy), δ 149.68 (2 C, 6,6′-arom. C from bipy), δ 148.62 (2 C, 3,3′-arom. C from bipy), δ 121.18 (2 C, 5,5′-arom. C from bipy).
  *MALDI-TOF-MS*: *m/z* 755.1 (M)^+^, *m/z* 373.1 ([Cr(dipic)(bipy)]^+^).
  *IR*: 3512.90 cm^−1^ hydrogen bonds, 1669.82 cm^−1^ C=O, 1602.69 cm^−1^ C-C (aromatic) stretching vibrations, 3151.61 cm^−1^ C-H (aromatic), 851.07 cm^−1^ C-N (aromatic), 1218.53 cm^−1^ O-C=O, 594.47 cm^−1^ Cr-O.
2. [Cr(dipic)_2_]Hdmbipy∙2.5 H_2_O

*UV-Vis*: The region of maximum absorption occurs at 550 nm. (in DMSO)

^1^*H NMR* (DMSO-d_6_, 500 MHz): δ 8.63 (ps, 2 H, 6,6′-arom. H from dmbipy), δ 8.17 (ps, 2 H, 3,3′-arom. H from dmbipy), δ 7.32 (ps, 2 H, 5,5′-arom. H from dmbipy), δ 4.04 (s, 6 H, O-CH_3_), δ 8.24 (ps, 4 H, 3, 5, 3′, 5′-arom. H from dipic), δ 8.17 (ps, 2 H, 4, 4′-arom. H from dipic), δ 1.23 (ps, 1 H, HN^+^-arom. from dipic).

^13^*C NMR* (DMSO-d_6_, 125 MHz): δ 169.05 (2 C, 2,2′-arom. C from dmbipy), δ 152.05 (2 C, 4,4′-arom. C from dmbipy), δ 149.22 (2 C, 6,6′-arom. C from dmbipy), δ 148.62 (2 C, 3,3′-arom. C from dmbipy), δ 112.66 (2 C, 5,5′-arom. C from dmbipy), 165.93 (4 C, C=O), δ 57.70 (2 C, O-CH_3_), δ 128.02 (4 C, 3, 5, 3′, 5′-arom. C from dipic), δ 128.02 (4 C, 3, 5, 3′, 5′-arom. C from dipic), δ 137.59 (2 C, 4, 4′-arom. C from dipic).

*MALDI-TOF-MS*: *m/z* 599.0 (M)^+^, *m/z* 569.3 (M minus two CH_3_- from dmbipy), *m/z* 555.9 (M minus COO group from dipic).

IR: 3431.90 cm^−1^ hydrogen bonds, 1666.97 cm^−1^ C=O, 1588.52 cm^−1^ C-C (aromatic) stretching vibrations, 3072.09 cm^−1^ C-H (aromatic), 839.64 cm^−1^ C-N (aromatic), 1258.95 cm^−1^ O-C=O, 581.66 cm^−1^ Cr-O.

### The polymerization of 2-chloro-2-propen-1-ol

**Figure 3:**
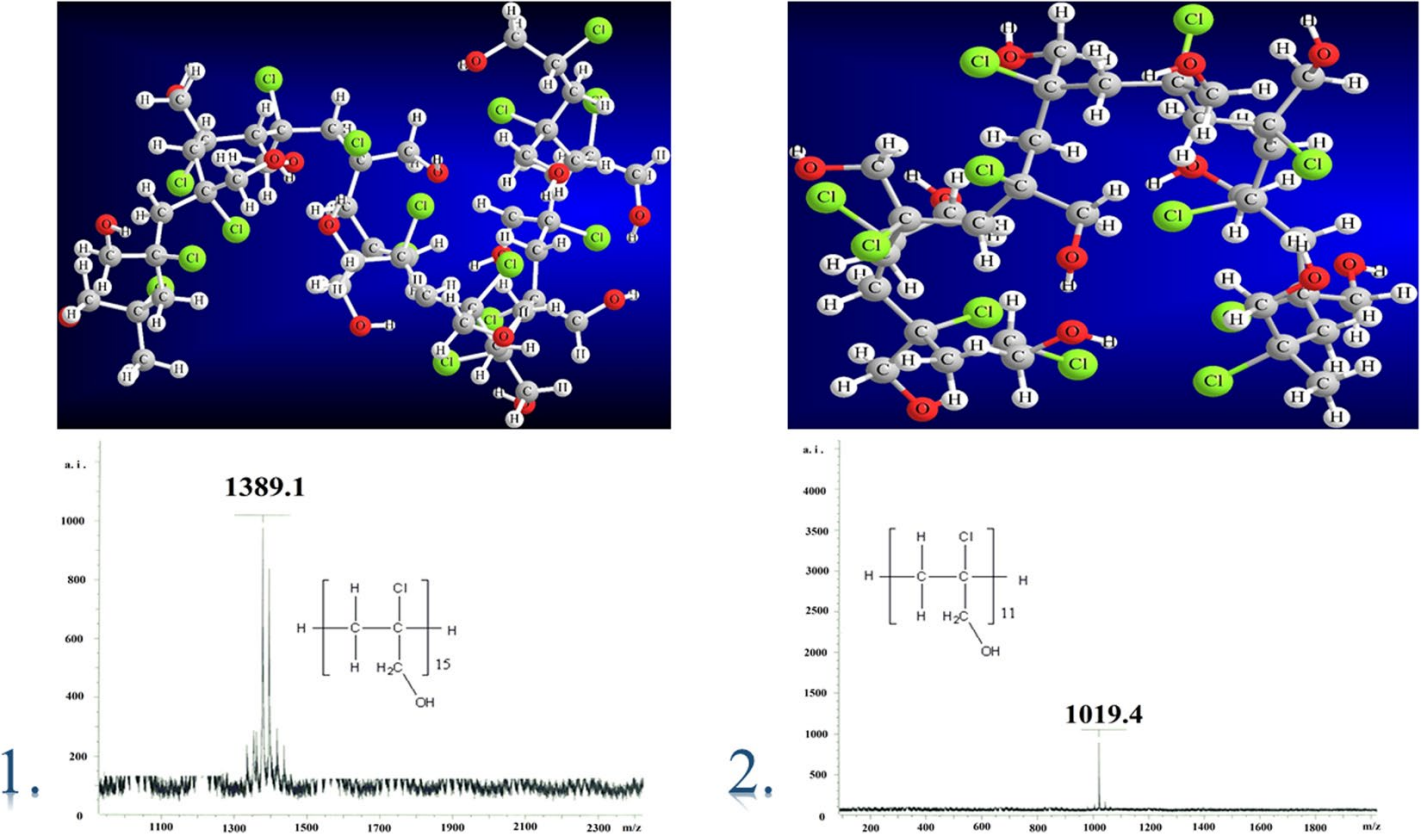
The MS spectra for the products of 2-chloro-2-propen-1-ol polymerization: (1) the polymer consisting of 15 monomers, (2) the polymer consisting of 11 monomers.

The products of polymerization process were analyzed by the MS, ^1^H and ^13^C NMR methods. It has been found that [Cr(dipic)_2_][Cr(bipy)(dipic)H_2_O]∙2 H_2_O as catalyst causes that the polymer consisting of 15 monomers (1389.5 g/mol) is formed while the second catalyst - [Cr(dipic)_2_]Hdmbipy∙2.5 H_2_O contributed to the formation of the polymer having the molecular mass equal to 1019.5 g/mol. MS spectra confirmed the values of molecular masses of two polymers (Fig. 3). In the catalytic system the MS spectra show two oligomers for the system with [Cr(dipic)_2_][Cr(bipy)(dipic)H_2_O]∙2 H_2_O: the first contains 11 monomers (1021.1 m/z) and the second contains 13 monomers (1199.1 m/z). In the case of the system with [Cr(dipic)_2_]Hdmbipy∙2.5 H_2_O three oligomers occurs in the catalytic system. These oligomers contain 5, 7 and 9 monomers with the values 469.3, 665.3 and 843.3 m/z, respectively.

**Figure 4:**
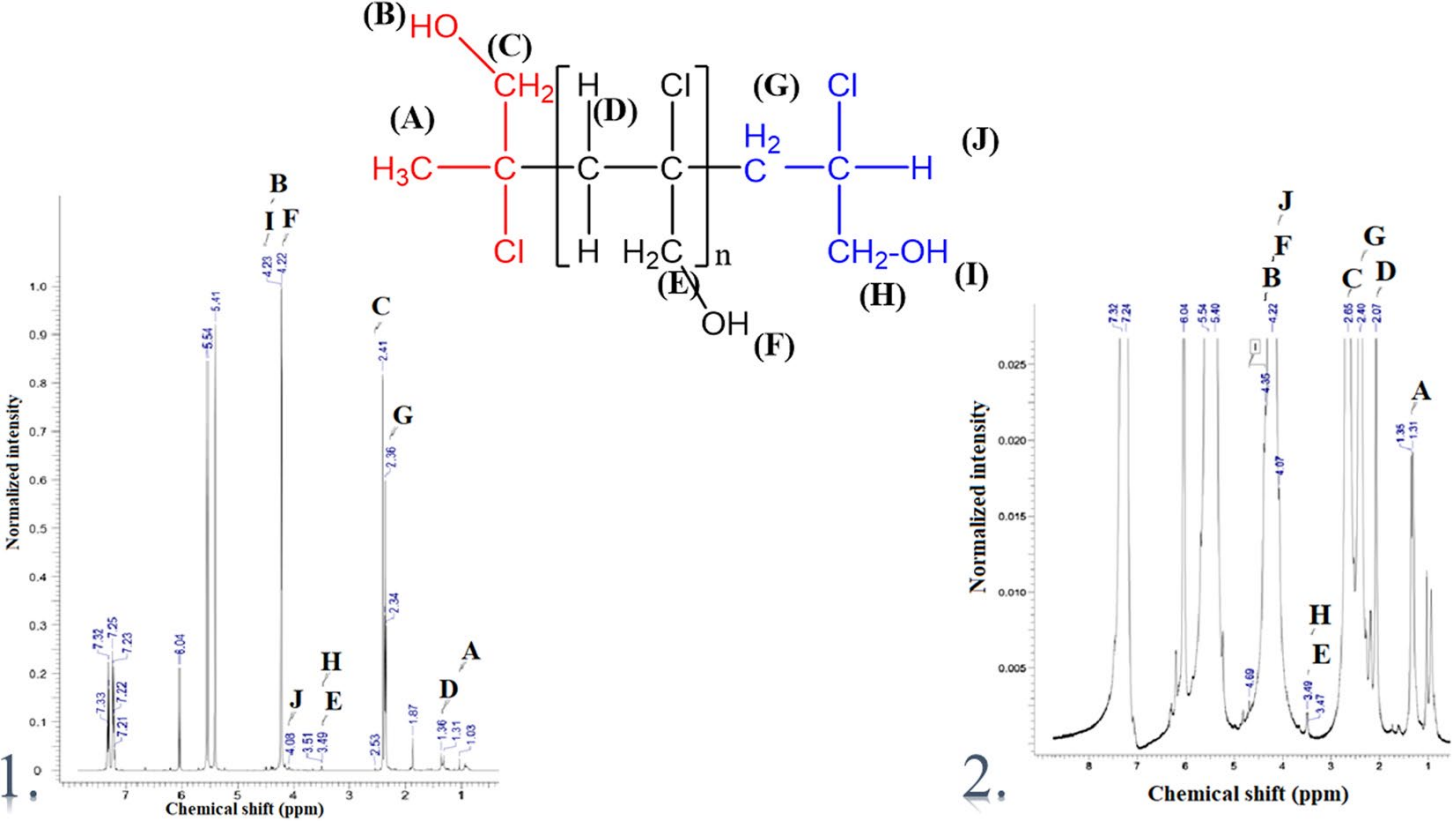
The ^1^H NMR spectra for systems: (1) the polymer of 2-chloro-2-propen-1-ol (15 monomers), [Cr(dipic)_2_][Cr(bipy)(dipic)H_2_O]∙2H_2_O and MMAO-12; (2) the polymer of 2-chloro-2-propen-1-ol (11 monomers), [Cr(dipic)_2_]Hdmbipy∙2.5H_2_O and MMAO-12.

**Figure 5:**
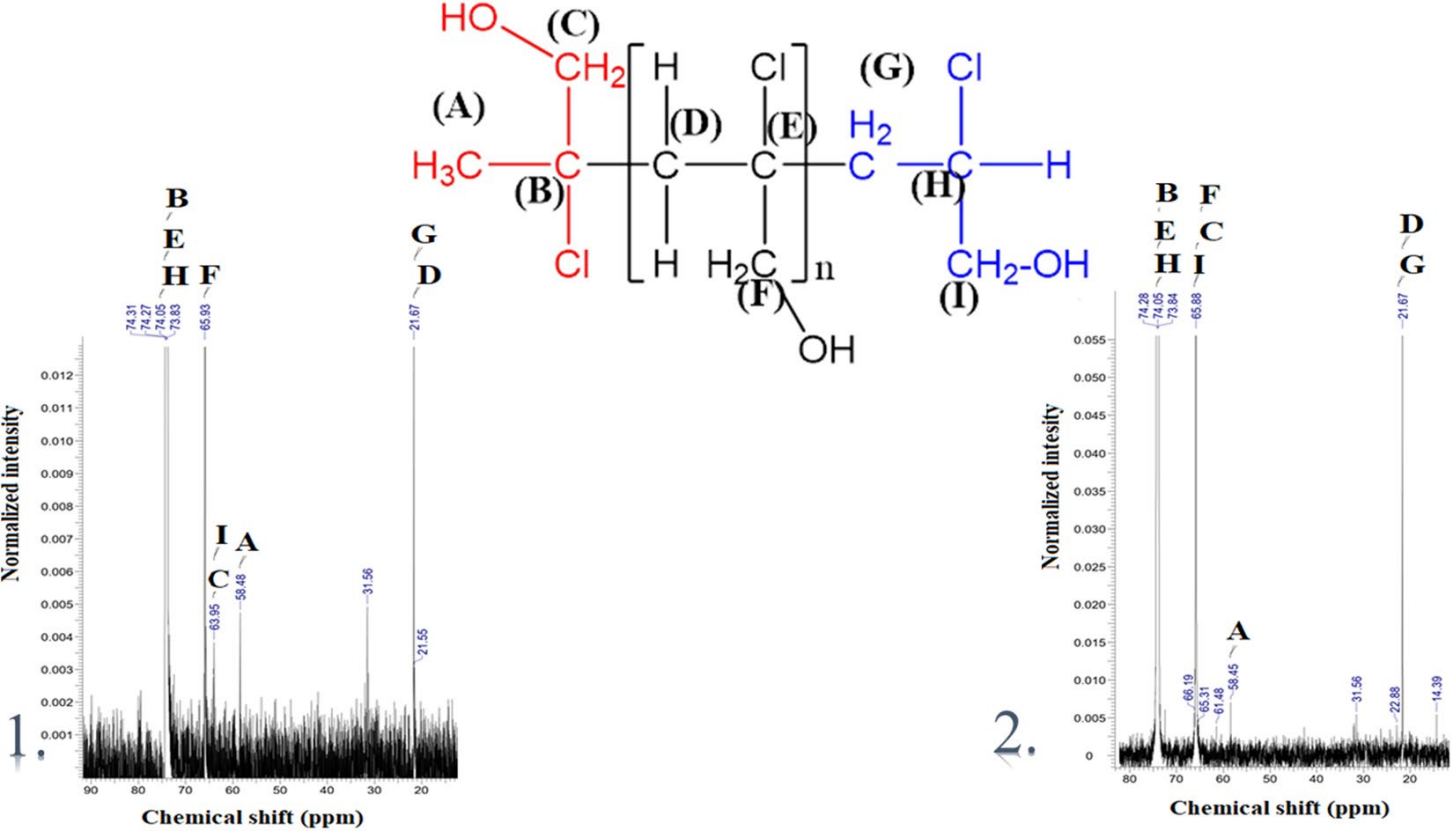
The ^13^C NMR spectra for systems: (1) the polymer of 2-chloro-2-propen-1-ol (15 monomers), [Cr(dipic)_2_][Cr(bipy)(dipic)H_2_O]∙2 H_2_O and MMAO-12; (2) the polymer of 2-chloro-2-propen-1-ol (11 monomers), [Cr(dipic)_2_]Hdmbipy∙2.5H_2_O and MMAO-12.

In addition, the ^1^H and ^13^C NMR spectra allowed the verification of the polymers compositions (Figs 4 and 5).

The polymerization results are summarized in Table 1, which includes representative data.

**Table 1:** Data for the polymerization of 2-chloro-2-propen-1-ol using chromium(III) complexes and MMAO-12.

Entry	Compound	Amt of Cr (µmol)	t (min)	Molar ratio compound:MMAO	Amt of polymer (g)	Activity (g∙mmol^−1^∙h^−1^)
1	[Cr(dipic)_2_][Cr(bipy) (dipic)H_2_O]∙2H_2_O	3	45	1:1000	5.95	2609.86
2	[Cr(dipic)_2_]Hdmbipy ∙2,5H_2_O	3	45	1:1000	5.14	2254.57

## Discussion

[Cr(dipic)_2_][Cr(bipy)(dipic)H_2_O]∙2H_2_O and [Cr(dipic)_2_]Hdmbipy∙2.5H_2_O complexes after activation by MMAO-12 have become remarkably active catalysts for the polymerization of 2-chloro-2-propen-1-ol. NMR spectroscopy revealed the tactic nature of both polymers. The analysis of the ^13^C NMR spectra of both the complexes ([Cr(dipic)_2_][Cr(bipy)(dipic)H_2_O]∙2H_2_O and [Cr(dipic)_2_]Hdmbipy∙2.5 H_2_O) and the polymers have confirmed that the small number of signals in the range of 20 ppm–75 ppm are the effect of the presence of isotactic polymers^18,19^.

[Cr(dipic)_2_][Cr(bipy)(dipic)H_2_O]∙2H_2_O complex with MMAO-12 exhibit a higher catalytic activity - about 16% in comparison to the catalytic activity of system including [Cr(dipic)_2_]Hdmbipy∙2.5 H_2_O. These differences may result from the polymerization mechanisms. Another reason of the different catalytic activity of both complexes may be fact that one mol of [Cr(dipic)_2_][Cr(bipy)(dipic)H_2_O]∙2H_2_O molecules contain 2 moles of Cr (III). Thus, more moles of the active catalysts are formed during the complex activation by MMAO-12.

In order to compare the activity of the designed catalysts described in this report with others so far known in the literature, we have compiled activities of several non-metallocene chromium(III) complexes in Table 2. The comparison of their catalytic activities with the activity of our catalysts is quite difficult due to the fact that polyethylene was obtained using catalysts collected in Table 2 and due to other polymerization conditions (temperature and pressure). However, in this Table 2 data on catalysts having a relatively high catalytic activity are collected. All chromium(III) complexes summarized in Table 2 have inorganic anions with the exception of compound 4. They exhibit minimum about 2.6 and maximum 24 times lower catalytic activity than the novel catalysts ([Cr(dipic)_2_][Cr(bipy)(dipic)H_2_O]∙2H_2_O and [Cr(dipic)_2_]Hdmbipy ∙2,5H_2_O). Moreover, in the case of a comparison of the catalytic activity of most of the known catalysts used for the polymerization of various monomers, eg ethene, norbornene, hexene, the catalysts described in this work show more than 5 times higher catalytic activity^12,20–22^.

**Table 2:** The comparison of catalytic activities of various non-metallocene chromium(III) complexes in the ethylene polymerization.

Compound	Temperature [°C]	Pressure [bar]	Activity (g∙mmol^−1^∙h^−1^ ∙bar^−1^)	References
[Cr{tris(*N*-methylimidazol-2-yl)methoxymethane}Cl_3_]	100	40	208	^32^
[Cr{2-[2-(diphenylphosphino)-1-(N-methylimidazol-2-yl)ethyl]-N-methylimidazole}Cl_3_]	100	40	108	^32^
[Cr(1,3,5-triazacyclohexane)]Cl_3_	40	1	717	^25^
[(2,6-Me_2_Ph)_2_(nacnac)Cr (OEt_2_)CH_2_SiMe_3_] B(3,5-(CF_3_)_2_C_6_H_3_)_4_ (nacnac = 2,4-pentane-N,N’-bis(aryl)ketiminato)	75	3	228	^33^
[2,6-bis(imino)pyridyl]CrCl_3_	70	4	1000	^34^
CrMe[N(SiMe_2_CH_2_PPh_2_)_2_	300	206	500	^3^

The catalytic activities of NNN-coordinated Cr(III) complexes^23,24^ have been compared with the catalytic activities of the chromium(III) complexes reported in this work. The α,α′-bis(arylimino)-2,3:5,6-bis(pentamethylene)pyridyl-chromium(III) chlorides (where aryl = 2,6-Me_2_Ph **Cr1**, 2,6-Et_2_Ph **Cr2**, 2,6-i-Pr_2_Ph **Cr3**, 2,4,6-Me_3_Ph **Cr4**, 2,6-Et_2_-4-MePh **Cr5**) are NNN-coordinated complexes of chromium(III) and they exhibit the catalytic activities (after the use of MMAO as the co-catalyst) for ethylene polymerization in the range 3.73∙10^5^–160.27∙10^5^ g of polyethylene (mol of Cr)^−1^∙h^−1 ^^23^. After the conversion of these values of the catalytic activity to the values expressed in the units presented in this work (Table 2), the NNN-coordinated Cr(III) complexes have the catalytic activities in the range of 37.3–1602.7 g∙mmol^−1^∙h^−1^∙bar^−1^. When comparing them with the activities of the catalysts described in this work, it can be concluded that ONO-coordinated Cr(III) complexes such as [Cr(dipic)_2_][Cr(bipy)(dipic)H_2_O]∙2H_2_O and [Cr(dipic)_2_]Hdmbipy∙2,5H_2_O have minimum about 1.4 and maximum 70 times higher catalytic activity than the NNN- coordinated complexes of chromium(III) catalysts. Moreover, in the case of NNN-coordinated Cr(III) complexes the 2 times higher amount of MMAO as the precatalyst has to be used in the comparison with the amount used in this work. Other examples of NNN- coordinated complexes of chromium(III) are [2-(ArNCMe)-8-(NAr)C_9_H_8_N]CrCl_3_ (Ar = 2,6-Me_2_Ph: **Cr1**; 2,6-Et_2_Ph: **Cr2**; 2,6-*i*Pr_2_Ph: **Cr3**; 2,4,6-Me_3_Ph: **Cr4**; 2,6-Et_2_-4-MePh: **Cr5**). These complexes have similar catalytic activities for ethylene polymerization, in the range 1.07 ∙ 10^6^–15.96 ∙ 10^6^ g of polyethylene (mol of Cr (III))^−1^∙h^−1 ^^24^, as the above mentioned NNN-coordinated Cr(III) complexes^23^.

## Conclusions

This work reports the new type of catalysts in the field of polymerization of beta-olefin derivatives. The catalysts constitute chromium(III) salt type complexes composed of both organic cations and anions. The high catalytic activity of two new complexes [Cr(dipic)_2_][Cr(bipy)(dipic)H_2_O]∙2H_2_O and [Cr(dipic)_2_]Hdmbipy∙2.5H_2_O activated by MMAO-12 have been studied towards the 2-chloro-2-propen-1-ol polymerization. This polymerization undergoes very easily at the room temperature and at atmospheric pressure. In this work we have shown that the studied non-metallocene complexes catalyze the formation of two polymers of 2-chloroallyl alcohol, namely polymers consisting of 15 monomers and 11 monomers in the case of [Cr(dipic)_2_][Cr(bipy)(dipic)H_2_O]∙2H_2_O and [Cr(dipic)_2_]Hdmbipy∙2.5H_2_O, respectively. The both new catalysts exhibit more than 5 times higher catalytic activity in comparison with the chromium(III) complexes containing bis(imino)pyridine^21^, imine-, alkoxy- and alkylthio-substituted cyclopentadienyl groups^22^ and triazacyclohexane^25^, which have been used for the ethylene or propylene polymerization.

## Methods

### Materials

All reagents were purchased from Sigma-Aldrich: 2,6-pyridinedicarboxylic acid (H_2_dipic, 99% purity), 2,2′-bypiridine (bipy, 98% purity), 4,4′-dimethoxy-2,2′-bipyridine (dmbipy, 97% purity), chromium trichloride hexahydrate (96%), toluene (99% purity), modified methylaluminoxane (MMAO-12, 7 wt % aluminum in toluene), 2-chloro-2-propen-1-ol (90%).

### Synthesis

The synthesis of [Cr(dipic)_2_][Cr(bipy)(dipic)H_2_O]∙2 H_2_O has been conducted according to the following procedure. To the aqueous solution containing CrCl_3_ ∙ 6 H_2_O (2.0 mmol, 0.54 g) the ethanolic solution of 2,6-pyridinedicarboxylic acid (2.0 mmol, 0.34 g) was added while keeping continuous stirring (a magnetic stirrer). Next to this mixture the ethanolic solution of 2,2′-bipyridine (2.2 mmol, 0.34 g) was added. In the next step the system was heated for about 15 minutes at 50 °C in a heating mantle. After cooling the mixture of trichloromethane (5 mL) and methanol (10 mL) was added. Then, after four days, the violet crystals of [Cr(dipic)_2_][Cr(bipy)(dipic)H_2_O]∙2 H_2_O were obtained. The yield was 79%. [Cr(dipic)_2_]Hdmbipy ∙ 2.5 H_2_O was prepared according to the same procedure as [Cr(dipic)_2_][Cr(bipy)(dipic)H_2_O]∙2 H_2_O, however, instead of the 2,2′-bipyridine solution was used the solution of 4,4′-dimethoxy-2,2′-bipyridine (2.2 mmol, 0.48 g) in ethanol. The yield was 65%.

The composition of the studied compounds was determined on the basis of the elemental analysis of carbon, hydrogen and nitrogen (CARBO ERBA type CHNS – O 1108). Anal. Calcd for [Cr(dipic)_2_][Cr(bipy)(dipic)H_2_O]∙2 H_2_O (%): C, 45.95, H, 2.84, N, 8.65. Found: C, 45.88, H, 2.75, N, 8.42. Anal. Calcd for [Cr(dipic)_2_]Hdmbipy ∙ 2.5 H_2_O (%): C, 48.41, H, 3.72, N, 8.69. Found: C, 48.25, H, 3.56, N, 8.57.

### X-Ray measurements

Single-crystal samples of [Cr(dipic)_2_][Cr(bipy)(dipic)H_2_O]∙2H_2_O and [Cr(dipic)_2_]Hdmbipy∙2.5H_2_O were chosen for the X-ray diffraction measurements at 295.2 K (see Tables in Supplementary Material). Data were collected using the Oxford Diffraction Gemini R ULTRA Ruby CCD diffractometer with the Mo *Kα* (λ = 0.71073 Å) radiation. The structural determination was performed using the SHELX package. The lattice parameters were determined based on a least-squares fit to the optimized setting angles of the collected reflections by means of CrysAlis CCD^26^. The structures were determined using direct methods, with refinements being carried out by full-matrix least-squares on *F*^2^ using the SHELXL-97 program^27^. H-atoms bound to N-atoms were located on a difference Fourier map and refined with restraints (DFIX command) where U_iso_(H) = 1.2U_eq_(N). H atoms from the water molecules were detected on a difference Fourier map and refined with restraints using DFIX command where U_iso_(H) = 1.5U_eq_(O). The occupancy factor for the disordered water molecules was 0.5. C–H atoms were detected in a difference map and were refined as riding with the distance constraints: C–H = 0.93 Å and with U_iso_(H) = 1.2 U_eq_(C) (C–H = 0.93 Å and with U_iso_(H) = 1.2 U_eq_(C) for methyl groups). The computational material - the all interactions demonstrated were revealed with the PLATON program^28^. The all graphics for publication were made using the PLUTO-78, ORTEPII and Mercury programs^29–31^.

Full crystallographic details, excluding structure features, have been deposited (deposition No. CCDC 1586740 and 1586741) for title compounds with the Cambridge Crystallographic Data Center. These data may be obtained, on request, from The Director, CCDC, 12 Union Road, Cambridge, CB2 1EZ, UK (e-mail: deposit@ccdc.cam.ac.uk or http://www.ccdc.cam.ac.uk).

### IR spectra

The IR spectra were recorded over the 4000–650 cm^−1^ range in a KBr pellet using the BRUKER IFS 66 spectrophotometer.

### UV-Vis spectra

The UV-Vis spectra were recorded using the Perkin-Elmer Lambda 650 instrument supplied with the temperature control (Peltier System) with a scan accuracy of 1 nm and a 1 nm slit width at a scanning rate 120.00 nm.min^−1^ (298 K). The title complexes were dissolved in DMSO. The concentration of each complex was 5 mM.

### NMR spectra

The ^1^H and ^13^C NMR spectra (C_2_D_2_Cl_4_ – for polymers and DMSO-d_6_ – for complexes) were obtained using the Bruker Avance III 500 (500.13/125.76 MHz) instrument (300 K).

### MS spectra

The positive-ion mode MALDI-TOF mass spectra were recorded on the Bruker Biflex III spectrometer with 2, 5 -dihydroxybenzoic acid (DHB) matrixes.

### The polymerization process

All studies were conducted under the nitrogen atmosphere at 21 °C and at atmospheric pressure. The violet solution of [Cr(dipic)_2_][Cr(bipy)(dipic)H_2_O]∙2 H_2_O (1.5 μmol, 1.2 mg) in toluene (2 mL) was added to the glass cell with a sealed stopper using a glass syringe. Then 3 mL of MMAO-12 solution was added into the glass cell. The mixture changed color to brown. The solutions in the cell were mixed all the time (a magnetic stirrer). Then 2-chloro-2-propen-1-ol was added dropwise. After 45 minutes the sticky light yellow gel was obtained.

The second polymer was obtained by the same procedure, however, [Cr(dipic)_2_]Hdmbipy ∙ 2.5 H_2_O (3.0 μmol, 1.9 mg) was used as the catalyst.

## Electronic supplementary material

Crystallography data of the new compounds
