# Supplementary material for: New type of highly active chromium(III) catalysts containing both organic cations and anions designed for polymerization of beta-olefin derivatives
Source: Sci Rep. 2018 Feb 2;8:2315. doi: 10.1038/s41598-018-20665-x (PMC5797135; doi:10.1038/s41598-018-20665-x)
Supplement: Supplementary file 1 — Crystallography data of the new compounds [file 41598_2018_20665_MOESM1_ESM.pdf]

New type of highly active chromium(III) catalysts containing both organic cations and anions designed for polymerization of beta-olefin derivatives

Joanna Drzeżdżon*, Artur Sikorski, Lech Chmurzyński, Dagmara Jacewicz

Table 1 Crystal data and structure refinement for title compounds.

Chemical formula	$[Cr(dipic)_2]Hdmbipy \cdot 2.5\ H_2O$	$[Cr(dipic)_2][Cr(bipy)(dipic)H_2O] \cdot 2\ H_2O$
FW/g mol^{-1}	1288.98	809.54
Crystal system	monoclinic	triclinic
Space group	*I*2/a	*P*-1
a/Å	18.223(3)	8.407(2)
b/Å	10.422(2)	14.316(6)
c/Å	29.482(6)	14.806(6)
α/°	90	102.22(3)
β/°	91.41(2)	102.83(3)
γ/°	90	103.64(4)
V/Å^3	5597.5(18)	1621.6(12)
Z	4	2
T/K	295(2)	295(2)
λ_{Mo}/Å	0.71073	0.71073
ρ_{calc}/g cm^{-3}	1.530	1.658
μ/mm^{-1}	0.483	0.754
F(000)	2656	824
θ range for data collection/º	3.51 – 25.00	3.32 – 25.00
Completeness 2θ /%	99.7	99.5
Reflections collected	17418	10935
Reflections unique	4917 [R_{int}=0.0495]	5663 [R_{int}=0.0495]
Data/restraints/parameters	4917 /8/430	5663 /12/500
Goodness-of-fit on F^2	1.022	0.925
Final R_1 value ($I > 2\sigma(I)$)	0.0518	0.0712
Final wR_2 value ($I > 2\sigma(I)$)	0.1362	0.1329
Final R_1 value (all data)	0.0739	0.1850
Final wR_2 value (all data)	0.1510	0.1907
CCDC number	1586740	1586741

Table 2 Bond lengths (Å) and angles (°) for $[Cr(dipic)_2]Hdmbipy \cdot 2.5\ H_2O$.

**O(1W)-H(1WA)**	**0.86(2)**
**O(2W)-H(2WA)**	**0.87(2)**
**O(2W)-H(2WB)**	**0.87(2)**
**O(3W)-H(3WA)**	**0.9833**
**O(3W)-H(3WB)**	**0.6417**
**O(3WA)-H(3WC)**	**0.8856**
**O(3WA)-H(3WD)**	**0.8008**

Cr(1)-N(1)	1.970(2)
Cr(1)-N(13)	1.972(2)
Cr(1)-O(20)	1.976(2)
Cr(1)-O(9)	1.988(2)
Cr(1)-O(11)	1.989(2)
Cr(1)-O(24)	1.995(2)
N(1)-C(2)	1.329(4)
N(1)-C(6)	1.331(4)
C(2)-C(3)	1.383(4)
C(2)-C(7)	1.517(4)
C(3)-C(4)	1.382(5)
C(3)-H(3)	0.9300
C(4)-C(5)	1.392(5)
C(4)-H(4)	0.9300
C(5)-C(6)	1.383(4)
C(5)-H(5)	0.9300
C(6)-C(10)	1.513(4)
C(7)-O(8)	1.223(4)
C(7)-O(9)	1.299(3)
C(10)-O(12)	1.219(4)
C(10)-O(11)	1.295(3)
N(13)-C(14)	1.327(4)
N(13)-C(18)	1.336(4)
C(14)-C(15)	1.388(4)
C(14)-C(19)	1.508(4)
C(15)-C(16)	1.388(5)
C(15)-H(15)	0.9300
C(16)-C(17)	1.384(5)
C(16)-H(16)	0.9300
C(17)-C(18)	1.382(4)
C(17)-H(17)	0.9300
C(18)-C(22)	1.515(5)
C(19)-O(21)	1.217(4)
C(19)-O(20)	1.299(4)
C(22)-O(23)	1.219(4)
C(22)-O(24)	1.291(4)
N(25)-C(26)	1.326(6)
N(25)-C(30)	1.334(4)
C(26)-C(27)	1.372(6)
C(26)-H(26)	0.9300
C(27)-C(28)	1.393(6)
C(27)-H(27)	0.9300
C(28)-O(37)	1.341(5)
C(28)-C(29)	1.376(5)

C(29)-C(30)	1.395(5)
C(29)-H(29)	0.9300
C(30)-C(31)	1.469(5)
C(31)-C(36)	1.367(5)
C(31)-N(32)	1.375(4)
N(32)-C(33)	1.331(5)
N(32)-H(32)	0.868(19)
C(33)-C(34)	1.347(6)
C(33)-H(33)	0.9300
C(34)-C(35)	1.396(5)
C(34)-H(34)	0.9300
C(35)-O(39)	1.324(5)
C(35)-C(36)	1.396(5)
C(36)-H(36)	0.9300
O(37)-C(38)	1.293(9)
O(37)-C(38A)	1.397(8)
C(38)-H(38C)	0.9600
C(38)-H(38B)	0.9600
C(38)-H(38A)	0.9600
C(38A)-C(38A)#1	1.868(17)
C(38A)-H(38F)	0.9600
C(38A)-H(38E)	0.9600
C(38A)-H(38D)	0.9600
O(39)-C(40)	1.422(5)
C(40)-H(40C)	0.9600
C(40)-H(40B)	0.9600
C(40)-H(40A)	0.9600

H(2WA)-O(2W)-H(2WB)	92(5)
H(3WA)-O(3W)-H(3WB)	114.6
H(3WC)-O(3WA)-H(3WD)	172.8
N(1)-Cr(1)-N(13)	176.83(10)
N(1)-Cr(1)-O(20)	97.85(9)
N(13)-Cr(1)-O(20)	79.05(9)
N(1)-Cr(1)-O(9)	78.92(9)
N(13)-Cr(1)-O(9)	101.78(9)
O(20)-Cr(1)-O(9)	93.05(9)
N(1)-Cr(1)-O(11)	79.05(9)
N(13)-Cr(1)-O(11)	100.28(9)
O(20)-Cr(1)-O(11)	91.09(9)
O(9)-Cr(1)-O(11)	157.93(8)
N(1)-Cr(1)-O(24)	104.30(9)
N(13)-Cr(1)-O(24)	78.80(9)
O(20)-Cr(1)-O(24)	157.84(9)

O(9)-Cr(1)-O(24)	92.05(9)
O(11)-Cr(1)-O(24)	92.24(9)
C(2)-N(1)-C(6)	123.4(2)
C(2)-N(1)-Cr(1)	118.52(19)
C(6)-N(1)-Cr(1)	118.07(18)
N(1)-C(2)-C(3)	119.8(3)
N(1)-C(2)-C(7)	111.3(2)
C(3)-C(2)-C(7)	129.0(3)
C(4)-C(3)-C(2)	118.3(3)
C(4)-C(3)-H(3)	120.8
C(2)-C(3)-H(3)	120.8
C(3)-C(4)-C(5)	120.7(3)
C(3)-C(4)-H(4)	119.7
C(5)-C(4)-H(4)	119.7
C(6)-C(5)-C(4)	118.1(3)
C(6)-C(5)-H(5)	121.0
C(4)-C(5)-H(5)	121.0
N(1)-C(6)-C(5)	119.7(3)
N(1)-C(6)-C(10)	111.5(2)
C(5)-C(6)-C(10)	128.8(3)
O(8)-C(7)-O(9)	125.2(3)
O(8)-C(7)-C(2)	121.4(3)
O(9)-C(7)-C(2)	113.4(3)
C(7)-O(9)-Cr(1)	117.89(18)
O(12)-C(10)-O(11)	125.3(3)
O(12)-C(10)-C(6)	121.1(3)
O(11)-C(10)-C(6)	113.6(3)
C(10)-O(11)-Cr(1)	117.71(18)
C(14)-N(13)-C(18)	123.6(3)
C(14)-N(13)-Cr(1)	118.0(2)
C(18)-N(13)-Cr(1)	118.4(2)
N(13)-C(14)-C(15)	119.8(3)
N(13)-C(14)-C(19)	111.5(3)
C(15)-C(14)-C(19)	128.7(3)
C(16)-C(15)-C(14)	117.8(3)
C(16)-C(15)-H(15)	121.1
C(14)-C(15)-H(15)	121.1
C(17)-C(16)-C(15)	121.2(3)
C(17)-C(16)-H(16)	119.4
C(15)-C(16)-H(16)	119.4
C(18)-C(17)-C(16)	118.3(3)
C(18)-C(17)-H(17)	120.9
C(16)-C(17)-H(17)	120.9
N(13)-C(18)-C(17)	119.4(3)

N(13)-C(18)-C(22)	111.1(2)
C(17)-C(18)-C(22)	129.4(3)
O(21)-C(19)-O(20)	125.2(3)
O(21)-C(19)-C(14)	121.3(3)
O(20)-C(19)-C(14)	113.4(3)
C(19)-O(20)-Cr(1)	117.9(2)
O(23)-C(22)-O(24)	125.6(3)
O(23)-C(22)-C(18)	120.6(3)
O(24)-C(22)-C(18)	113.8(3)
C(22)-O(24)-Cr(1)	117.9(2)
C(26)-N(25)-C(30)	116.0(4)
N(25)-C(26)-C(27)	125.1(4)
N(25)-C(26)-H(26)	117.4
C(27)-C(26)-H(26)	117.4
C(26)-C(27)-C(28)	118.4(4)
C(26)-C(27)-H(27)	120.8
C(28)-C(27)-H(27)	120.8
O(37)-C(28)-C(29)	121.4(4)
O(37)-C(28)-C(27)	120.7(4)
C(29)-C(28)-C(27)	117.9(4)
C(28)-C(29)-C(30)	118.9(3)
C(28)-C(29)-H(29)	120.6
C(30)-C(29)-H(29)	120.6
N(25)-C(30)-C(29)	123.7(3)
N(25)-C(30)-C(31)	114.2(3)
C(29)-C(30)-C(31)	122.1(3)
C(36)-C(31)-N(32)	117.4(3)
C(36)-C(31)-C(30)	126.4(3)
N(32)-C(31)-C(30)	116.2(3)
C(33)-N(32)-C(31)	122.2(3)
C(33)-N(32)-H(32)	134(3)
C(31)-N(32)-H(32)	104(3)
N(32)-C(33)-C(34)	121.9(3)
N(32)-C(33)-H(33)	119.1
C(34)-C(33)-H(33)	119.1
C(33)-C(34)-C(35)	118.5(4)
C(33)-C(34)-H(34)	120.7
C(35)-C(34)-H(34)	120.7
O(39)-C(35)-C(34)	124.0(4)
O(39)-C(35)-C(36)	117.0(3)
C(34)-C(35)-C(36)	118.9(4)
C(31)-C(36)-C(35)	120.9(3)
C(31)-C(36)-H(36)	119.6
C(35)-C(36)-H(36)	119.6

**C(38)-O(37)-C(28)**	**125.0(6)**
**C(38)-O(37)-C(38A)**	**105.9(7)**
**C(28)-O(37)-C(38A)**	**127.0(4)**
**O(37)-C(38)-H(38C)**	**109.5**
**O(37)-C(38)-H(38B)**	**109.5**
**H(38C)-C(38)-H(38B)**	**109.5**
**O(37)-C(38)-H(38A)**	**109.5**
**H(38C)-C(38)-H(38A)**	**109.5**
**H(38B)-C(38)-H(38A)**	**109.5**
**O(37)-C(38A)-C(38A)#1**	**177.8(9)**
**O(37)-C(38A)-H(38F)**	**109.5**
**C(38A)#1-C(38A)-H(38F)**	**69.0**
**O(37)-C(38A)-H(38E)**	**109.5**
**C(38A)#1-C(38A)-H(38E)**	**70.0**
**H(38F)-C(38A)-H(38E)**	**109.5**
**O(37)-C(38A)-H(38D)**	**109.5**
**C(38A)#1-C(38A)-H(38D)**	**72.7**
**H(38F)-C(38A)-H(38D)**	**109.5**
**H(38E)-C(38A)-H(38D)**	**109.5**
**C(35)-O(39)-C(40)**	**118.9(3)**
**O(39)-C(40)-H(40C)**	**109.5**
**O(39)-C(40)-H(40B)**	**109.5**
**H(40C)-C(40)-H(40B)**	**109.5**
**O(39)-C(40)-H(40A)**	**109.5**
**H(40C)-C(40)-H(40A)**	**109.5**
**H(40B)-C(40)-H(40A)**	**109.5**

Symmetry transformations used to generate equivalent atoms:
#1 -x+1/2,y,-z+1

Table 3

Bond lengths (Å) and angles (°) for $[Cr(dipic)_2][Cr(bipy)(dipic)H_2O] \cdot 2\ H_2O$

**O(1W)-Cr(2)**	**1.949(5)**
**O(1W)-H(1WA)**	**0.85(2)**
**O(1W)-H(1WB)**	**0.86(2)**
**O(2W)-H(2WA)**	**0.84(2)**
**O(2W)-H(2WB)**	**0.84(2)**
**O(3W)-H(3WA)**	**0.8026**
**O(3W)-H(3WB)**	**0.8517**
**O(3W)-H(3WD)**	**1.2786**
**O(3WA)-H(3WC)**	**0.9094**

O(3WA)-H(3WD)	0.7873
Cr(1)-N(13)	1.964(6)
Cr(1)-N(1)	1.965(5)
Cr(1)-O(24)	1.974(5)
Cr(1)-O(20)	1.977(5)
Cr(1)-O(11)	1.992(5)
Cr(1)-O(9)	2.003(5)
N(1)-C(2)	1.331(8)
N(1)-C(6)	1.333(9)
Cr(2)-O(32)	1.965(5)
Cr(2)-N(25)	1.979(5)
Cr(2)-O(36)	2.000(5)
Cr(2)-N(44)	2.035(6)
Cr(2)-N(37)	2.056(6)
C(2)-C(3)	1.377(9)
C(2)-C(7)	1.500(10)
C(3)-C(4)	1.365(10)
C(3)-H(3)	0.9300
C(4)-C(5)	1.394(10)
C(4)-H(4)	0.9300
C(5)-C(6)	1.394(9)
C(5)-H(5)	0.9300
C(6)-C(10)	1.513(10)
C(7)-O(8)	1.227(8)
C(7)-O(9)	1.287(7)
C(10)-O(12)	1.229(9)
C(10)-O(11)	1.300(9)
N(13)-C(18)	1.326(8)
N(13)-C(14)	1.346(9)
C(14)-C(15)	1.376(10)
C(14)-C(19)	1.530(11)
C(15)-C(16)	1.366(11)
C(15)-H(15)	0.9300
C(16)-C(17)	1.394(11)
C(16)-H(16)	0.9300
C(17)-C(18)	1.376(9)
C(17)-H(17)	0.9300
C(18)-C(22)	1.511(11)
C(19)-O(21)	1.200(10)
C(19)-O(20)	1.304(10)
C(22)-O(23)	1.211(8)
C(22)-O(24)	1.321(8)
N(25)-C(30)	1.323(9)
N(25)-C(26)	1.341(9)

C(26)-C(27)	1.390(9)
C(26)-C(31)	1.514(11)
C(27)-C(28)	1.396(11)
C(27)-H(27)	0.9300
C(28)-C(29)	1.378(10)
C(28)-H(28)	0.9300
C(29)-C(30)	1.379(9)
C(29)-H(29)	0.9300
C(30)-C(34)	1.500(10)
C(31)-O(33)	1.219(9)
C(31)-O(32)	1.316(8)
C(34)-O(35)	1.239(9)
C(34)-O(36)	1.300(8)
N(37)-C(38)	1.325(8)
N(37)-C(42)	1.363(8)
C(38)-C(39)	1.396(9)
C(38)-H(38)	0.9300
C(39)-C(40)	1.398(10)
C(39)-H(39)	0.9300
C(40)-C(41)	1.358(10)
C(40)-H(40)	0.9300
C(41)-C(42)	1.379(9)
C(41)-H(41)	0.9300
C(42)-C(43)	1.457(10)
C(43)-N(44)	1.365(8)
C(43)-C(48)	1.401(10)
N(44)-C(45)	1.306(10)
C(45)-C(46)	1.388(11)
C(45)-H(45)	0.9300
C(46)-C(47)	1.373(12)
C(46)-H(46)	0.9300
C(47)-C(48)	1.368(12)
C(47)-H(47)	0.9300
C(48)-H(48)	0.9300

Cr(2)-O(1W)-H(1WA)	127(5)
Cr(2)-O(1W)-H(1WB)	123(5)
H(1WA)-O(1W)-H(1WB)	109(3)
H(2WA)-O(2W)-H(2WB)	115(3)
H(3WA)-O(3W)-H(3WB)	120.1
H(3WA)-O(3W)-H(3WD)	101.8
H(3WB)-O(3W)-H(3WD)	105.9
H(3WC)-O(3WA)-H(3WD)	135.0
N(13)-Cr(1)-N(1)	177.9(3)

N(13)-Cr(1)-O(24)	79.0(2)
N(1)-Cr(1)-O(24)	98.9(2)
N(13)-Cr(1)-O(20)	80.2(2)
N(1)-Cr(1)-O(20)	101.9(2)
O(24)-Cr(1)-O(20)	159.2(2)
N(13)-Cr(1)-O(11)	100.6(2)
N(1)-Cr(1)-O(11)	79.3(2)
O(24)-Cr(1)-O(11)	90.5(2)
O(20)-Cr(1)-O(11)	93.6(2)
N(13)-Cr(1)-O(9)	101.4(2)
N(1)-Cr(1)-O(9)	78.7(2)
O(24)-Cr(1)-O(9)	92.7(2)
O(20)-Cr(1)-O(9)	91.0(2)
O(11)-Cr(1)-O(9)	158.0(2)
C(2)-N(1)-C(6)	123.5(6)
C(2)-N(1)-Cr(1)	118.3(5)
C(6)-N(1)-Cr(1)	118.1(5)
O(1W)-Cr(2)-O(32)	94.2(2)
O(1W)-Cr(2)-N(25)	92.3(2)
O(32)-Cr(2)-N(25)	79.3(2)
O(1W)-Cr(2)-O(36)	88.5(2)
O(32)-Cr(2)-O(36)	157.9(2)
N(25)-Cr(2)-O(36)	78.6(2)
O(1W)-Cr(2)-N(44)	169.4(2)
O(32)-Cr(2)-N(44)	92.1(2)
N(25)-Cr(2)-N(44)	97.2(3)
O(36)-Cr(2)-N(44)	88.9(2)
O(1W)-Cr(2)-N(37)	91.1(2)
O(32)-Cr(2)-N(37)	99.3(2)
N(25)-Cr(2)-N(37)	176.3(3)
O(36)-Cr(2)-N(37)	102.6(2)
N(44)-Cr(2)-N(37)	79.4(3)
N(1)-C(2)-C(3)	118.7(7)
N(1)-C(2)-C(7)	111.6(6)
C(3)-C(2)-C(7)	129.7(7)
C(4)-C(3)-C(2)	119.8(7)
C(4)-C(3)-H(3)	120.1
C(2)-C(3)-H(3)	120.1
C(3)-C(4)-C(5)	121.0(7)
C(3)-C(4)-H(4)	119.5
C(5)-C(4)-H(4)	119.5
C(4)-C(5)-C(6)	117.0(8)
C(4)-C(5)-H(5)	121.5
C(6)-C(5)-H(5)	121.5

N(1)-C(6)-C(5)	120.0(7)
N(1)-C(6)-C(10)	111.2(7)
C(5)-C(6)-C(10)	128.8(8)
O(8)-C(7)-O(9)	126.4(7)
O(8)-C(7)-C(2)	119.6(6)
O(9)-C(7)-C(2)	114.0(7)
C(7)-O(9)-Cr(1)	117.3(5)
O(12)-C(10)-O(11)	125.6(7)
O(12)-C(10)-C(6)	120.5(8)
O(11)-C(10)-C(6)	113.9(8)
C(10)-O(11)-Cr(1)	116.7(5)
C(18)-N(13)-C(14)	124.2(7)
C(18)-N(13)-Cr(1)	119.2(5)
C(14)-N(13)-Cr(1)	116.5(5)
N(13)-C(14)-C(15)	118.2(8)
N(13)-C(14)-C(19)	112.8(7)
C(15)-C(14)-C(19)	128.8(9)
C(16)-C(15)-C(14)	118.5(9)
C(16)-C(15)-H(15)	120.8
C(14)-C(15)-H(15)	120.8
C(15)-C(16)-C(17)	122.4(8)
C(15)-C(16)-H(16)	118.8
C(17)-C(16)-H(16)	118.8
C(18)-C(17)-C(16)	116.7(8)
C(18)-C(17)-H(17)	121.7
C(16)-C(17)-H(17)	121.7
N(13)-C(18)-C(17)	119.9(8)
N(13)-C(18)-C(22)	110.7(6)
C(17)-C(18)-C(22)	129.4(8)
O(21)-C(19)-O(20)	126.5(8)
O(21)-C(19)-C(14)	121.9(9)
O(20)-C(19)-C(14)	111.7(8)
C(19)-O(20)-Cr(1)	118.3(5)
O(23)-C(22)-O(24)	125.8(8)
O(23)-C(22)-C(18)	120.7(7)
O(24)-C(22)-C(18)	113.5(7)
C(22)-O(24)-Cr(1)	117.4(5)
C(30)-N(25)-C(26)	123.2(6)
C(30)-N(25)-Cr(2)	118.9(5)
C(26)-N(25)-Cr(2)	117.7(5)
N(25)-C(26)-C(27)	119.9(8)
N(25)-C(26)-C(31)	111.6(7)
C(27)-C(26)-C(31)	128.5(8)
C(26)-C(27)-C(28)	117.4(8)

C(26)-C(27)-H(27)	121.3
C(28)-C(27)-H(27)	121.3
C(29)-C(28)-C(27)	120.9(7)
C(29)-C(28)-H(28)	119.6
C(27)-C(28)-H(28)	119.6
C(28)-C(29)-C(30)	118.8(8)
C(28)-C(29)-H(29)	120.6
C(30)-C(29)-H(29)	120.6
N(25)-C(30)-C(29)	119.9(7)
N(25)-C(30)-C(34)	110.5(6)
C(29)-C(30)-C(34)	129.6(8)
O(33)-C(31)-O(32)	126.2(9)
O(33)-C(31)-C(26)	121.0(8)
O(32)-C(31)-C(26)	112.8(7)
C(31)-O(32)-Cr(2)	118.6(5)
O(35)-C(34)-O(36)	123.8(7)
O(35)-C(34)-C(30)	120.8(7)
O(36)-C(34)-C(30)	115.4(7)
C(34)-O(36)-Cr(2)	116.3(5)
C(38)-N(37)-C(42)	118.5(6)
C(38)-N(37)-Cr(2)	126.2(5)
C(42)-N(37)-Cr(2)	115.3(5)
N(37)-C(38)-C(39)	123.8(7)
N(37)-C(38)-H(38)	118.1
C(39)-C(38)-H(38)	118.1
C(38)-C(39)-C(40)	116.2(8)
C(38)-C(39)-H(39)	121.9
C(40)-C(39)-H(39)	121.9
C(41)-C(40)-C(39)	120.7(7)
C(41)-C(40)-H(40)	119.7
C(39)-C(40)-H(40)	119.7
C(40)-C(41)-C(42)	119.6(8)
C(40)-C(41)-H(41)	120.2
C(42)-C(41)-H(41)	120.2
N(37)-C(42)-C(41)	121.2(8)
N(37)-C(42)-C(43)	114.1(7)
C(41)-C(42)-C(43)	124.7(8)
N(44)-C(43)-C(48)	119.4(8)
N(44)-C(43)-C(42)	116.1(7)
C(48)-C(43)-C(42)	124.6(8)
C(45)-N(44)-C(43)	119.4(7)
C(45)-N(44)-Cr(2)	125.9(6)
C(43)-N(44)-Cr(2)	114.7(5)
N(44)-C(45)-C(46)	123.6(9)

**N(44)-C(45)-H(45)**	**118.2**
**C(46)-C(45)-H(45)**	**118.2**
**C(47)-C(46)-C(45)**	**117.7(9)**
**C(47)-C(46)-H(46)**	**121.1**
**C(45)-C(46)-H(46)**	**121.1**
**C(48)-C(47)-C(46)**	**119.7(9)**
**C(48)-C(47)-H(47)**	**120.2**
**C(46)-C(47)-H(47)**	**120.2**
**C(47)-C(48)-C(43)**	**119.9(9)**
**C(47)-C(48)-H(48)**	**120.0**
**C(43)-C(48)-H(48)**	**120.0**

Table 4 Hydrogen bonding interactions in the crystal structure of Cr(dipic)$_2$]Hdmbipy · 2.5 H_2O.

D–H···A	*d*(D–H) (Å)	*d*(H···A) (Å)	*d*(D···A) (Å)	<D–H···A (°)
O1WA–H1WA···O2B^{i}	0.81(3)	1.86(3)	2.656(3)	172(3)
O1WA–H2WA···O2C^{ii}	0.77(3)	1.84(3)	2.603(3)	170(3)
O1WB–H2WB···O1A^{iii}	0.83(3)	2.28(3)	3.058(3)	155(3)
O1WB–H2WB···O3A^{iii}	0.83(3)	2.41(3)	3.039(3)	134(3)
O1WB–H1WB···O2A	0.82(3)	2.24(3)	2.973(3)	149(3)
N10–H10···O2A	0.85(3)	2.01(3)	2.735(4)	143(3)
C2A–H2A···N1	0.97	2.62	3.520(5)	154
C2A–H2B···O3A^{iii}	0.97	2.32	3.271(4)	167
C4–H4···O3A^{iv}	0.93	2.58	3.261(4)	130
C5–H5···O1WB^{v}	0.93	2.50	3.288(4)	142
C7–H7···O2C^{vi}	0.93	2.30	3.224(4)	170
C8–H8···O1A^{vi}	0.93	2.51	3.171(4)	128

Symmetry code: (i) 1/2–x, 1/2+y, 3/2–z; (ii) 1/2–x, –1/2+y, 3/2–z; (iii) 1+x, y, z; (iv) 1+x, 1+y, z; (v) 2–x, 1–y, 1–z; (vi) 1–x, 1–y, 1–z.

Table 5 Hydrogen bonding interactions in the crystal structure of

[Cr(dipic)$_2$][Cr(bipy)(dipic)H_2O] · 2 H_2O

D–H···A	*d*(D–H) (Å)	*d*(H···A) (Å)	*d*(D···A) (Å)	<D–H···A (°)
O1W–H1WA···O2W	0.85(5)	1.66(5)	2.502(9)	169(9)
O1W–H2WA···O8^{i}	0.85(6)	1.86(6)	2.645(8)	153(8)
O2WB–H2WA···O35^{ii}	0.84(6)	1.90(6)	2.728(8)	172(6)
O2WB–H2WB···O24^{iii}	0.84(6)	1.87(6)	2.707(8)	174(6)
O3WB–H3WA···O21	0.80(6)	2.04(6)	2.630(8)	130(9)

O3WB–H3WB···O11^{iv}	0.85(6)	2.05(6)	2.900(8)	174(9)
C3–H3···O8^{v}	0.93	2.39	3.121(9)	1335
C4–H4···O2W^{vi}	0.93	2.59	3.436(9)	151
C5–H5···O35^{vii}	0.93	2.41	3.281(9)	155
C16–H16···O32^{ivi}	0.93	2.49	3.141(9)	127
C17–H17··· O36^{viii}	0.93	2.50	3.269(9)	140
C28–H28··· O20^{vi}	0.93	2.47	3.349(9)	157
C29–H29··· O12^{vii}	0.93	2.54	3.4089(9)	155
C38–H38···O1W	0.93	2.49	3.016(9)	116
C47–H47···O12	0.93	2.60	3.002(9)	107
C48–H48···O3W^{ix}	0.93	2.46	3.370(9)	167

Symmetry code: (i) –1+x, –1+y, z; (ii) 1+x, y, z; (iii) x, –1+y, z; (iv) 1–x, 1–y, 1–z; (v) 2–x, 2–y, 2–z; (vi) 1–x, 1–y, 2–z; (vii) –x, 1–y, 2–z; (viii) –x, 1–y,1–z; (ix) –1+x, y, z.
